# DASAF: An R Package for Deep Sequencing-Based Detection of Fetal Autosomal Abnormalities from Maternal Cell-Free DNA

**DOI:** 10.1155/2016/2714341

**Published:** 2016-06-29

**Authors:** Baohong Liu, Xiaoyan Tang, Feng Qiu, Chunmei Tao, Junhui Gao, Mengmeng Ma, Tingyan Zhong, JianPing Cai, Yixue Li, Guohui Ding

**Affiliations:** ^1^State Key Laboratory of Veterinary Etiological Biology, Lanzhou Veterinary Research Institute, Chinese Academy of Agricultural Sciences, Xujiaping 1, Lanzhou, Gansu 730046, China; ^2^Key Laboratory of Systems Biology, Institute of Biochemistry and Cell Biology, Shanghai Institutes for Biological Sciences, Chinese Academy of Sciences, Shanghai 200031, China; ^3^Shanghai Center for Bioinformation Technology, Shanghai Industrial Technology Institute, Shanghai 200235, China; ^4^EG Information Technology Enterprise (EGI), Basepair Biotechonology. Co. Ltd., Suzhou 215123, China

## Abstract

*Background*. With the development of massively parallel sequencing (MPS), noninvasive prenatal diagnosis using maternal cell-free DNA is fast becoming the preferred method of fetal chromosomal abnormality detection, due to its inherent high accuracy and low risk. Typically, MPS data is parsed to calculate a risk score, which is used to predict whether a fetal chromosome is normal or not. Although there are several highly sensitive and specific MPS data-parsing algorithms, there are currently no tools that implement these methods.* Results*. We developed an R package, detection of autosomal abnormalities for fetus (DASAF), that implements the three most popular trisomy detection methods—the standard *Z*-score (STDZ) method, the GC correction *Z*-score (GCCZ) method, and the internal reference *Z*-score (IRZ) method—together with one subchromosome abnormality identification method (SCAZ).* Conclusions*. With the cost of DNA sequencing declining and with advances in personalized medicine, the demand for noninvasive prenatal testing will undoubtedly increase, which will in turn trigger an increase in the tools available for subsequent analysis. DASAF is a user-friendly tool, implemented in R, that supports identification of whole-chromosome as well as subchromosome abnormalities, based on maternal cell-free DNA sequencing data after genome mapping.

## 1. Introduction

Fetal autosomal aneuploidies are one type of abnormalities for chromosome number with a death rate of 6%–11% in newborns. And the most common autosomal aneuploidies are Down's syndrome (trisomy 21) with the incidence of 1 in every 160 newborns causing mental retardation and hypoplasia [1]. Besides whole-chromosome aneuploidies, a considerable number of fetuses are at high risk for subchromosomal abnormalities [2–4] that also result in mental illnesses and other abnormalities [5].

The traditional screening for chromosomal abnormalities combined the maternal age, ultrasonographic examination of the fetus, and levels of various proteins or hormones in the maternal blood which refers to traditional noninvasive detection [6]. However, the traditional noninvasive methods are lacking accuracy because they are indirect measures of the underlying chromosomal defect [7, 8]. So pregnant women have to choose the invasive methods including chorionic villus sampling and cordocentesis, coupled with fetal cell karyotyping which yield definitive answers. But there is a 0.5% risk of miscarriage which adds additional concern to the pregnant women and their families [9, 10].

The discovery of cell-free fetal DNA in maternal serum [11] and recent advances in massive parallel sequencing (MPS) technologies [12–14] now enable noninvasive prenatal testing (NIPT) of fetal chromosomal aneuploidies [15–18], with very high specificity and sensitivity [19–21]. In addition to being noninvasive, NIPT requires only 5 mL of maternal peripheral blood for sequencing. Sequences are analyzed using bioinformatics methods to calculate a hazard score, which is then used to determine whether fetal chromosomes are normal or not. Although the standard *Z*-score (STDZ) method was originally used, it was later discovered that the accuracy of this method varied depending on the GC content of the chromosomes in question [15]. More specifically, the coefficients of variance (of measuring the percentage of representation of each chromosome) were much larger for chromosomes 18 and 13 than for chromosome 21 [15, 16]. This variation in accuracy is linked to the difference in sequencing efficacy as a function of chromosome size and GC content. In recent years, many methods have emerged to solve the aforementioned problem, including a GC correction *Z*-score (GCCZ) method [21], internal reference *Z*-score (IRZ) method [20], and the noninvasive fetal trisomy (NIFTY) test [19], as well as the method of Srinivasan et al., henceforth referred to as the subchromosome abnormality *Z*-score (SCAZ) method [22]. The first three methods are similar to the standard method (*Z*-score) for identifying abnormalities in whole chromosomes, while the last method is used to identify subchromosomal (i.e., chromosomal regions) losses and gains. Lau et al. indicated that the standard *Z*-score (STDZ) method accurately detects trisomy 21 early in pregnancy of 11 weeks with low accuracy for other aneuploidies, being 0% for trisomy 13 and 40% for trisomy 18, while the GC correction with LOESS regression method (GCCZ) is more accurate than STDZ but still with low detection rate for trisomy 18. And the adjusted method using *Z*-scores with an internal reference (IRZ), which corrects for GC bias and sequencing efficiency, substantially improved the performance of the test [20].

On the other hand, Verweij et al. investigated the attitudes among pregnant women regarding NIPT for the detection of trisomy 21 (T21): they had a positive attitude regarding NIPT for detection of T21, and more than 50% of them who rejected the traditional screening would accept NIPT if available [23]. However, although NIPT has become increasingly popular and acceptable and subsequent data analysis algorithms have emerged, there are no tools currently available to implement these data analysis methods. In the present study, we developed an R package, DASAF, that implements the three most popular trisomy detection methods (STDZ, GCCZ, and IRZ) and one subchromosome abnormality identification method (SCAZ). We have also included a fetal gender prediction module in the DASAF package. With the cost of DNA sequencing declining and with advances in personalized medicine, we believe that the demand for NIPT will increase, which will undoubtedly trigger an increase in the tools available for subsequent analysis.

## 2. Materials and Methods

This study was approved by the Independent Ethics Committee of Shanghai Clinical Research Center. The reference data used here consists of DNA sequencing data from one hundred and twenty pregnant women from Huzhou Maternity & Child Care Hospital located in Huzhou, Zhejiang, China. All data were produced by Illumina HiSeq2000 for 100 bp pair-end with 7 × 10^6^ to 17 × 10^6^ sequence read pairs per sample.

The sequencing reads were aligned to the human genome assembly hg19 with Bowtie short read aligner (version 1.1.2), allowing for two base mismatches at most when aligning [24]. Only uniquely mapped reads were kept.

**Figure 1:**
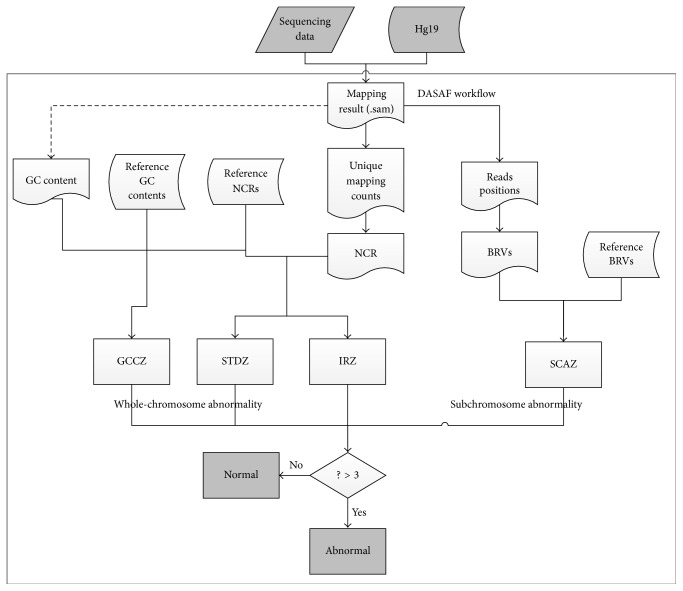
Workflow of the R package DASAF. The DASAF workflow consists of two main parts: (a) mapping reads statistics and (b) autosomal aneuploidy prediction. The results from (a) are used to calculate the risk score using any of the four methods implemented in (b). STDZ: standard *Z*-score; GCCZ: GC correction *Z*-score; IRZ: internal reference *Z*-score; and SCAZ: subchromosome abnormalities *Z*-score.

Before using DASAF, sequencing data should be aligned using the above method, which is independent of the DASAF software and needs to be completed by the users themselves. The results file from Bowtie is used as input for DASAF. All the reference datasets are described in Table 1. A typical DASAF workflow involves two procedures: mapping read statistics and autosomal aneuploidy prediction (Figure 1).

### 2.1. Read Mapping Statistics

Read mapping statistics produce two files: one contains the unique mapping read counts for every chromosome and the other contains the mapping location for every unique read. The normalized chromosome ratio (NCR) is generated according to the following equation for every chromosome in each sample: NCR is the ratio of number of reads uniquely mapped to the specific chromosome divided by the total number of reads uniquely mapped to all autosomal chromosomes [15, 25]. If the GCCZ method is used, the GC content for every chromosome is calculated from the mapping results.

### 2.2. Autosomal Aneuploidy Prediction

#### 2.2.1. Standard *Z*-Score Method

In the standard *Z*-score (STDZ) theory method, a hazard ratio of the *Z*-score is calculated to determine whether the fetal chromosome is normal or not:

(1) $$\begin{array}{c} {\left(\;STDZ\;\right)}_{i}=\frac{NCR_{i}-{\overset{-}{NCR}}_{i}}{SD_{i}}, \end{array}$$

where NCR_*i*_ is the ratio of the sequence counts uniquely mapped to the specific chromosome and the total number of the sequences uniquely mapped to all of the autosomal chromosomes, ${\overset{-}{NCR}}_{i}$ is the average NCR of chromosome *i* in the reference samples, SD_*i*_ is the standard deviation for NCRs of chromosome *i* in the reference samples, and *i* is the specific chromosome number, that is, 13, 18, and 21 [15].

For the average value and standard deviation values for the NCRs, one can use the reference files (NCR_ref.txt) contained in the DASAF package or calculate them based on one's own samples. The *Z*-score is a number indicating how far an observation deviates from the average in a population [26]. Usually, a *Z*-score of 3 is selected as threshold to determine whether the fetus is normal or not [22].

#### 2.2.2. GC Correction *Z*-Score Method

We calculated the slope from the NCR values (in reference file NCR_ref.txt) of chromosomes 13, 18, and 21 of the 120 reference samples against their GC content (in reference file GC_ref.txt) by linear regression and a corrected NCR value will be calculated using the following equation:

(2) $$\begin{array}{c} NCR_{GC}=NCR-\frac{\left(GC-GC_{average\_ref}\right)}{Slope_{ref}}, \end{array}$$

where NCR_GC_ is the NCR value after GC correction, NCR is the original value, and GC_average_ref_ and Slope_ref_ are the mean values of references' chromosomal GC content and the slope of linear regression from the reference samples [21].

Then, the mean and SD of the GC-corrected NCR were calculated for the reference dataset and the *Z*-score was calculated for the chromosome of the sample tested using (1) with a *Z*-score cutoff of 3.

#### 2.2.3. Internal Reference *Z*-Score Method

To minimize the sequencing bias (stemming from differences in GC content), Lau et al. presented a *Z*-score method that relies on an internal reference chromosome [20]. They showed that using chromosomes 4, 8, and 14 as internal reference chromosomes provided the most accurate results for the detection of trisomy 13, trisomy 18, and trisomy 21, respectively. The method is as follows: the comparative NCR is calculated using the value from the internal reference as NCR_*i*_/NCR_IR_, where IR is the internal reference chromosome for chromosome *i*. The *Z*-score is also calculated by (1) that the IR adjusted NCR value for the test sample subtracts the averaged IR adjusted NCR values from the reference samples and the difference is then divided by the standard deviation from the IR adjusted NCR values for the reference samples. A *Z*-score of 3 was selected as threshold for the diagnosis of trisomy in chromosome *i* of the testing sample [20].

#### 2.2.4. Subchromosome Abnormality *Z*-Score Method

In addition to whole-chromosome abnormalities, subchromosome losses and gains are also important components of chromosomal diseases [4]. The subchromosome abnormality *Z*-score (SCAZ) is a method used to identify abnormalities for chromosomal regions with lengths between 100 kb and 1 Mb [22]. In the first step, positions uniquely mapped to the genome are retrieved and counted as tags. And the whole genome was divided into continuous bins with length of 1 Mb and 100 kb and tags were assigned to individual bins for the following analysis. Then GC content percentage of each bin was calculated to rank the bins across the entire genome. And then every bin was normalized using the ratio of tags within the bin to the sum of the tag counts in bins with the nearest GC content percentages. Bins with nearest GC content percentages include 10 bins of 1 Mb length and 40 bins of 100 kb length. The equation is as follows:

(3) $$\begin{array}{c} BRV_{ij}=\frac{Tags_{ij}}{\sum Tags_{km}}, \end{array}$$

where BRV_*ij*_ is the ratio for the *j*th bin for chromosome *i* and Tags_*ij*_ is the count of tags in the *j*th bin for chromosome *i*. *km* represents the bins with length of 100 kb and 1 Mb.

Further, every BRV was examined for deviation from the median values collected across all the reference samples which is similar to the standard *Z*-score method, while the median absolute deviations (MAD) were adjusted to *a*MAD (i.e., MAD was multiplied by 1.4826); here *a* is 1.4826. Consider

(4) $$\begin{array}{c} {\left(\;SCAZ\;\right)}_{ij}=\frac{BRV_{ij}-mBRV_{ij}}{aMAD_{ij}}. \end{array}$$

The absolute values of *Z*-score larger than 3 indicate that there were CNVs in fetal chromosome for the specific genomic regions [22].

## 3. Results and Discussions

We built the DASAF R package, which supports three existing methods for identifying whole chromosome abnormalities and one for identifying subchromosome abnormalities from MPS data. We then compared the running time and identification accuracy of the four methods.

### 3.1. Comparison of Chromosomal Abnormality Detection Methods

All the detection methods used here were derived from existing algorithms and their accuracy has been tested previously [19, 20]. Here, we therefore only list the previously reported results for these algorithms. Lau et al. provided detection rates and false-positive rates for the three whole-chromosome trisomy detection methods. Their research revealed that the false-positive rates were 0 for all the three methods and the method of IRZ was the most sensitive, with a 100% detection rate for all trisomies examined (13, 18, and 21). For the method of STDZ, the detection rate was 100% for detecting trisomy 21 but only 40% for trisomy 18 and almost 0% for trisomy 13, while the GCCZ method with a detection rate of 100% for trisomy 21, 90% for trisomy 18, and 100% for trisomy 13 was better than the standard method but worse than the IRZ method [20]. Jiang et al. also evaluated the performance of these three methods for 903 cases and found that the Coefficient of Variation (CV) for the STDZ method was larger than that for the other two approaches among clinically relevant chromosomes (13, 18, and 21). Thus, the STDZ method has poor sensitivity for the detection of trisomies 13 and 18. However, the performance of the GCCZ approach demonstrated over 99% sensitivity and specificity for the detection of trisomies 13, 18, and 21, while the IRZ approach displayed CV larger than GCCZ but smaller than STDZ for chromosomal trisomies 13, 18, and 21 [19]. In summary, the adjusted methods (GCCZ and IRZ) more accurately identify trisomies than the STDZ method.

It was also reported that the SCAZ method, which identifies chromosome CNVs, can accurately detect losses and gains for chromosomal regions [22].

### 3.2. Evaluation of Diagnostic Accuracy as a Function of Sequencing Depth

**Figure 2:**
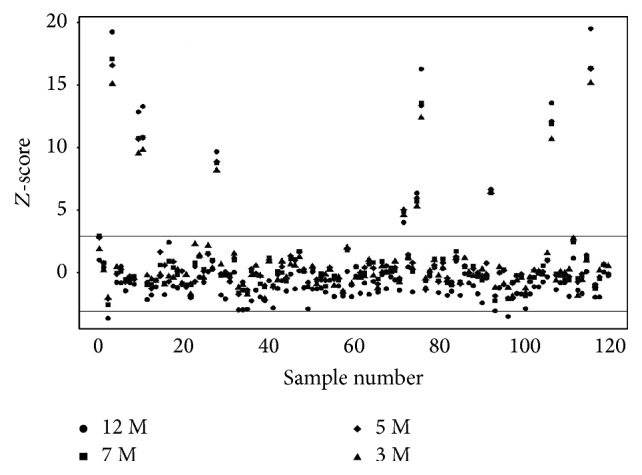
*Z*-score distribution for maternal cell-free DNA samples at varying sequencing depths. The black dots represent samples from the 100 bp pair-end run at a depth of 12 M reads. The black squares, diamonds, and triangles represent samples from the original 100 bp paired end run that have been randomly subsampled to 7 M, 5 M, and 3 M reads, respectively. The black line indicates the *Z*-score threshold of 3.

In order to evaluate the effect of sequencing depth on diagnostic accuracy, we randomly subsampled the 100 bp pair-end (PE) sequencing data at read counts of at least 3 M, 5 M, 7 M, and 12 M. Using the STDZ method, cases were diagnosed as T21-positive or T21-negative. Importantly, we found that, even at a read count of 3 M, T21 was accurately diagnosed, which suggests that the cost of sequencing can be considerably reduced by decreasing the sequence coverage. The results shown in Figure 2 demonstrate that the *Z*-scores for all the positive samples are larger that 3 (above the horizontal line of *y* = 3).

### 3.3. Execution Time Comparison

We tested the running time for all methods included in the DASAF package on datasets with read pairs of 12 M, 20 M, and 40 M (derived from patients of the Huzhou Maternity & Child Care Hospital).

STDZ and IRZ ran faster than the other methods if the NCR values for references were prepared beforehand. The GCCZ method requires the user to calculate the GC content for every chromosome, which consumes a considerable amount of time. The SCAZ method had the longest runtime because the BRV needs to be calculated for every bin by counting the tags. While all running times were acceptable, these times can be dramatically reduced by decreasing the sequence read counts to 3–5 M (Table 2).

## 4. Conclusions

We developed an R package that supports chromosomal abnormality detection. For chromosomal abnormality detection, users can select one of four supported methods or, for whole chromosomal abnormality detection, summarize the results of the three available methods (i.e., average the three *Z*-scores) for detection of trisomies 13, 18, and 21. We chose a *Z*-score threshold of 3 to predict fetal chromosome abnormalities. The reference datasets under the directory of data in the package can be updated or replaced by users as the samples increase, which can promote the accuracy of these methods. A detailed vignette is included with the DASAF package to assist nonexperts in the field (http://lifecenter.sgst.cn/dasaf/).

The cost of high-throughput sequencing has decreased dramatically over the past few years, thus increasing its utility in clinical practice [27, 28]. Noninvasive prenatal diagnosis is the most widely used method for detecting trisomic abnormalities or the loss or gain of chromosomal regions, and an increasing number of pregnant women are benefitting from this technology. In August 2014, noninvasive prenatal DNA diagnosis finally obtained legal status in China following the approval of the registration of second-generation gene-sequencing diagnostic products. This represents a major advance in the field of prenatal screening that will undoubtedly benefit numerous pregnant women and their families.

## Figures and Tables

**Table 1:** Description of reference datasets.

Dataset name	Description
NCR_ref	Ratios of uniquely mapping reads for every chromosome and the total number of sequences uniquely mapped to the genome for all 120 samples

GC_ref	GC content for every chromosome for all 120 samples

Tag_pos	Genomic bins positions for widths of 1 Mb and 100 kb

Bin_GC	GC content calculated for genomic bins for all samples

Nearest_bin_ref	Bin names of the 10 bins for the 1 Mb data and the 40 bins for the 100 kb data, which are with the nearest GC content for every divided bin

BRV_ref	Ratios of reads within a bin to the total number of reads in bins with the nearest GC percentages

**Table 2:** Execution time (in seconds) to detect chromosomal abnormalities using different methods.

	12 M reads	20 M reads	40 M reads
Standard *Z*-score (STDZ) method	1	1	2
GC Correction *Z*-score (GCCZ) method	312	629	1,156
Internal reference *Z*-score (IRZ) method	1	1	1
Subchromosome abnormality *Z*-score (SCAZ) method	2,074	2,105	2,278

The computing platform is a Linux system with 16 threads (0.8 GHZ for each) and RAM of 64 GB. Execution time was averaged over five repetitive runs.

## Abbreviations

DASAF: Detection of autosomal abnormalities for fetus
MPS: Massively parallel sequencing
NIPT: Noninvasive prenatal testing
STDZ: Standard *Z*-score
GCCZ: GC correction *Z*-score
IRZ: Internal reference *Z*-score
NIFTY: Noninvasive fetal trisomy
SCAZ: Subchromosome abnormalities *Z*-score
NCR: Normalized chromosome representation
BRV: Bin ratio value.

## Additional Points

(i) Project name is DASAF. (ii) Project home page is http://lifecenter.sgst.cn/dasaf/. (iii) Operating systems are Linux and Windows (Linux recommended). (iv) Programming languages are R and Perl. (vi) License is GPL ≥ 2. (vii) There is no nonacademics restrictions for using.

## References

[1] Driscoll D. A., Gross S. (2009). Prenatal screening for aneuploidy. *The New England Journal of Medicine*.

[2] Blumberg B. D., Shulkin J. D., Rotter J. I., Mohandas T., Kaback M. M. (1982). Minor chromosomal variants and major chromosomal anomalies in couples with recurrent abortion. *American Journal of Human Genetics*.

[3] Borgaonkar D. S., Bolling D. R., Partridge C., Ruddle F. H., McKusick V. A. (1975). Chromosomal variation in man: catalog of chromosomal variants and anomalies. *Birth Defects: Original Article Series*.

[4] Grati F. R., Barlocco A., Grimi B. (2010). Chromosome abnormalities investigated by non-invasive prenatal testing account for approximately 50% of fetal unbalances associated with relevant clinical phenotypes. *American Journal of Medical Genetics, Part A*.

[5] Bianchi D. W., Rava R. P., Sehnert A. J. (2014). DNA sequencing versus standard prenatal aneuploidy screening. *The New England Journal of Medicine*.

[6] Malone F. D., Canick J. A., Ball R. H. (2005). First-trimester or second-trimester screening, or both, for down's syndrome. *The New England Journal of Medicine*.

[7] Palomaki G. E., Hardow J. E., Knight G. J. (1995). Risk-based prenatal screening for trisomy 18 using alpha-fetoprotein, unconjugated oestriol and human chorionic gonadotropin. *Prenatal Diagnosis*.

[8] Crandall B. F., Hanson F. W., Keener S., Matsumoto M., Miller W. (1993). Maternal serum screening for *α*-fetoprotein, unconjugated estriol, and human chorionic gonadotropin between 11 and 15 weeks of pregnancy to detect fetal chromosome abnormalities. *American Journal of Obstetrics and Gynecology*.

[9] Odibo A. O., Dicke J. M., Gray D. L. (2008). Evaluating the rate and risk factors for fetal loss after chorionic villus sampling. *Obstetrics and Gynecology*.

[10] Odibo A. O., Gray D. L., Dicke J. M., Stamilio D. M., Macones G. A., Crane J. P. (2008). Revisiting the fetal loss rate after second-trimester genetic amniocentesis: a single center's 16-year experience. *Obstetrics & Gynecology*.

[11] Dennis Lo Y. M., Corbetta N., Chamberlain P. F. (1997). Presence of fetal DNA in maternal plasma and serum. *The Lancet*.

[12] Bentley D. R., Balasubramanian S., Swerdlow H. P. (2008). Accurate whole human genome sequencing using reversible terminator chemistry. *Nature*.

[13] Mardis E. R. (2011). A decade's perspective on DNA sequencing technology. *Nature*.

[14] Metzker M. L. (2010). Sequencing technologies the next generation. *Nature Reviews Genetics*.

[15] Chiu R. W. K., Chan K. C. A., Gao Y. (2008). Noninvasive prenatal diagnosis of fetal chromosomal aneuploidy by massively parallel genomic sequencing of DNA in maternal plasma. *Proceedings of the National Academy of Sciences of the United States of America*.

[16] Fan H. C., Blumenfeld Y. J., Chitkara U., Hudgins L., Quake S. R. (2008). Noninvasive diagnosis of fetal aneuploidy by shotgun sequencing DNA from maternal blood. *Proceedings of the National Academy of Sciences of the United States of America*.

[17] Chiu R. W. K., Sun H., Akolekar R. (2010). Maternal plasma DNA analysis with massively parallel sequencing by ligation for noninvasive prenatal diagnosis of trisomy 21. *Clinical Chemistry*.

[18] Lo Y. M. D. (2011). The quest for accurate measurement of fetal DNA in maternal plasma. *Clinical Chemistry*.

[19] Jiang F., Ren J., Chen F. (2012). Noninvasive Fetal Trisomy (NIFTY) test: an advanced noninvasive prenatal diagnosis methodology for fetal autosomal and sex chromosomal aneuploidies. *BMC Medical Genomics*.

[20] Lau T. K., Chen F., Pan X. (2012). Noninvasive prenatal diagnosis of common fetal chromosomal aneuploidies by maternal plasma DNA sequencing. *Journal of Maternal-Fetal and Neonatal Medicine*.

[21] Liang D., Lv W., Wang H. (2013). Non-invasive prenatal testing of fetal whole chromosome aneuploidy by massively parallel sequencing. *Prenatal Diagnosis*.

[22] Srinivasan A., Bianchi D. W., Huang H., Sehnert A. J., Rava R. P. (2013). Noninvasive detection of fetal subchromosome abnormalities via deep sequencing of maternal plasma. *American Journal of Human Genetics*.

[23] Verweij E. J., Oepkes D., de Vries M., van den Akker M. E., van den Akker E. S., de Boer M. A. (2013). Non-invasive prenatal screening for trisomy 21: what women want and are willing to pay. *Patient Education and Counseling*.

[24] Langmead B., Trapnell C., Pop M., Salzberg S. L. (2009). Ultrafast and memory-efficient alignment of short DNA sequences to the human genome. *Genome Biology*.

[25] Ehrich M., Deciu C., Zwiefelhofer T. (2011). Noninvasive detection of fetal trisomy 21 by sequencing of DNA in maternal blood: a study in a clinical setting. *American Journal of Obstetrics and Gynecology*.

[26] Sparks A. B., Struble C. A., Wang E. T., Song K., Oliphant A. (2012). Noninvasive prenatal detection and selective analysis of cell-free DNA obtained from maternal blood: evaluation for trisomy 21 and trisomy 18. *American Journal of Obstetrics and Gynecology*.

[27] Brownstein Z., Friedman L. M., Shahin H. (2011). Targeted genomic capture and massively parallel sequencing to identify genes for hereditary hearing loss in middle eastern families. *Genome Biology*.

[28] Bell C. J., Dinwiddie D. L., Miller N. A. (2011). Carrier testing for severe childhood recessive diseases by next-generation sequencing. *Science Translational Medicine*.

